# Epithelial sodium channel abundance is decreased by an unfolded protein response induced by hyperosmolality

**DOI:** 10.14814/phy2.12169

**Published:** 2014-11-20

**Authors:** Gilles Crambert, Thomas Ernandez, Christine Lamouroux, Isabelle Roth, Eva Dizin, Pierre‐Yves Martin, Eric Féraille, Udo Hasler

**Affiliations:** 1 UPMC/INSERM/Paris Descartes U1138 CNRS ERL 8228, Equipe 3 Métabolisme et Physiologie Rénale, Centre de Recherche des Cordeliers, Paris, France; 2 Department of Cellular Physiology and Metabolism and Service of Nephrology, University Medical Center, University of Geneva, Geneva, Switzerland

**Keywords:** Aquaporin‐2, endoplasmic reticulum stress, epithelial sodium channel, homeostasis, hyperosmolality, kidney collecting duct, unfolded protein response

## Abstract

Large shifts of osmolality occur in the kidney medulla as part of the urine concentrating mechanism. Hyperosmotic stress profoundly challenges cellular homeostasis and induces endoplasmic reticulum (ER) stress. Here, we examined the unfolded protein response (UPR) in hyperosmotically‐challenged principal cells of the kidney collecting duct (CD) and show its relevance in controlling epithelial sodium channel (ENaC) abundance, responsible for the final adjustment of Na^+^ excretion. Dehydration increases medullary but not cortical osmolality. Q‐PCR analysis of microdissected CD of water‐deprived mice revealed increased aquaporin‐2 (AQP2) expression in outer medullary and cortical CD while ENaC abundance decreased in outer medullary but not cortical CD. Immunoblotting, Q‐PCR and immunofluorescence revealed that hyperosmolality induced a transient ER stress‐like response both ex vivo and in cultured CD principal cells and increased activity of the canonical UPR mediators PERK and ATF6. Both hyperosmolality and chemical induction of ER stress decreased ENaC expression in vitro. ENaC depletion by either stimulus was abolished by transcriptional inhibition and by the chemical chaperone 4‐phenylbutyric acid and was partly abrogated by either PERK or ATF6 silencing. Our data suggest that induction of the UPR by hyperosmolality may help preserve body fluid homeostasis under conditions of dehydration by uncoupling AQP2 and ENaC abundance in outer medullary CD.

## Abstract (precis)

Hyperosmotic stress profoundly challenges cellular homeostasis and induces endoplasmic reticulum stress. We show that hyperosmolality triggers a unfolded protein response response in kidney collecting duct principal cells that decreases ENaC abundance. This may help preserve body fluid homeostasis under conditions of dehydration by uncoupling AQP2 and ENaC abundance in outer medullary CD.

## Introduction

Hyperosmolality damages cytosolic proteins, increases protein aggregation, alters membrane trafficking and induces autophagy (Carpentier et al. 1989; Heuser and Anderson 1989; Ellis and Minton 2006; Choe and Strange 2008; Hasler et al. 2008b; Munishkina et al. 2008; Zhou et al. 2008; Burkewitz et al. 2011; Nunes et al. 2013). These illustrate the dramatic and broad effects of hyperosmolality on cellular homeostasis and structural organization and offer the intriguing possibility that other major cellular processes might be affected. Endoplasmic reticulum (ER) stress is a fundamental stress response that allows cells to adapt to environmental change by restoring cellular homeostasis. It arises when the processing and folding capacities of the ER are overwhelmed, either by an overload of nascent proteins or by exogenous disruption of the protein folding and trafficking system (Rutkowski and Kaufman 2004; Ron and Walter 2007). The accumulation of unfolded protein in the ER lumen triggers the cytoprotective unfolded protein response (UPR) that transiently attenuates protein synthesis, degrades misfolded proteins and increases ER protein folding capacity. This allows cells to adapt to environmental change by restoring cellular homeostasis. If the stress persists and homeostasis cannot be reestablished, for instance when proteotoxic stimuli are excessive, UPR signaling can activate cell‐death pathways. In the kidney, extracellular osmolality increases along the corticomedullary axis and is enhanced by arginine vasopressin (AVP) in response to an increase of plasma osmolality (Sands and Layton 2014). Several pieces of experimental data have shown that alterations of inner medullary osmolality are accompanied by altered expression of genes that are typically induced by ER stress (Zhang et al. 1999; Tian and Cohen 2002; van Balkom et al. 2004; Hoorn et al. 2005; Cai et al. 2006, 2010). This strongly suggests that hypertonicity induces ER stress. How hyperosmolality affects UPR signaling is largely unexplored.

The final adjustment of salt and water excretion, critical for the regulation of whole body extracellular fluid volume and electrolyte balance, is achieved by the collecting duct (CD) and predominantly relies on epithelial sodium channel (ENaC, a heterotrimeric protein composed of *α*,* β* and *γ* subunits) and aquaporin‐2 (AQP2) expressed at the apical surface of principal cells. AVP increases both AQP2 abundance and its expression in the apical membrane (Fushimi et al. 1993; Nielsen et al. 1993). Accumulating evidence indicates that in addition to enhancing water transport AVP also stimulates Na^+^ reabsorption. Stimulation of Na^+^ reabsorption by aldosterone is synergized by AVP (Reif et al. 1986; Kudo et al. 1994; Verrey 1994). AVP increases Na^+^ transport by cortical CD (CCD) (Tomita et al. 1985; Nicco et al. 2001) and reduces sodium excretion (Bankir et al. 2005). The involvement of ENaC in this process is supported by the observation that both ENaC*β* and ENaC*γ* mRNA and protein abundance are increased by AVP in kidney cortex (Ecelbarger et al. 2000; Nicco et al. 2001). Furthermore, AVP increases ENaC activity in isolated CCD (Kudo et al. 1994; Nicco et al. 2001; Bugaj et al. 2009) and cultured CCD cells (Gaeggeler et al. 2011). Consistent with increased AQP2 and ENaC activities, transepithelial Na^+^ transport across CCD induced by AVP was found to be proportionally accompanied by osmotically‐driven water flow (Kudo et al. 1994; Nicco et al. 2001; Gaeggeler et al. 2011). This begs the question of how the kidney recalibrates water and salt transport under conditions of electrolyte imbalance, such as dehydration, which would necessitate uncoupling between water and salt transport. Interestingly, unlike AQP2, several studies have shown that expression levels of all three ENaC subunits globally decline from the cortex to the inner medulla (Duc et al. 1994; Vehaskari et al. 1998; MacDonald et al. 2000; Kim et al. 2004; Frindt et al. 2007), suggesting that coupled water and salt transport by CCD may not necessarily occur in medullary CD. The inverse relationship between ENaC abundance and the corticomedullary osmotic gradient suggests a role for hyperosmolality in decreased ENaC abundance. This is supported by the observation that abundance of at least some ENaC subunits in inner medullary CD (IMCD) are decreased by water restriction (Cai et al. 2006), which increases medullary osmolality. Inversely, ENaC levels are increased by vasopressin escape (Hoorn et al. 2005) and increased in IMCD of aquaporin‐1 knockout mice that display an impaired ability to concentrate urine (Morris et al. 2005). Possibly, hyperosmolality may decrease ENaC abundance by inducing ER stress.

In the present study, we examine how hyperosmolality affects UPR signaling and how this may affect ENaC abundance in outer medullary CD (OMCD), a tubule segment whose capacity to reabsorb water relies on extracellular osmolality. We show that water deprivation that increases extracellular osmolality of the medulla but not cortex decreases abundance of all three ENaC subunits in OMCD but not CCD while AQP2 abundance is increased in both cortical and medullary CD. Hyperosmolality produces similar effects in cultured mCCD_cl1_ and mpkCCD_cl4_ cells and induces an ER stress‐like response and UPR signaling in vitro and ex vivo that is linked with decreased ENaC abundance. Our study sheds new light on the mechanistic control of ENaC abundance in OMCD and provides evidence that hyperosmolality reinforces independent regulation of water and salt excretion by this tubule segment.

## Materials and Methods

### Materials

Antibodies are depicted in Table 1. Aldosterone, actinomycin D, and 4‐phenylbutyric acid were purchased from Sigma‐Aldrich (St. Louis, MO). Lipopolysaccharide, thapsigargin and tunicamycin were purchased from EMD Millipore (Billerica, MA).

**Table 1.** Antibodies

Primary IgG	Host	Dilution (WB)	Dilution (IF)	Manufacturer
Primary IgG				
AQP2	Rabbit	1:10000		Nielsen et al. (2006)
ENaC*α*	Rabbit	1:1000		Picard et al. (2008)
Tubulin	Mouse	1:10000		Sigma‐Aldrich, St. Louis, MO
Actin	Mouse	1:40000		Sigma‐Aldrich
GAPDH	Mouse	1:100000		Millipore, Billerica, MA
GRP78	Rabbit	1:1000		Abcam, Cambridge, UK
ATF4	Rabbit	1:1000	1:200	Cell Signaling, Danvers, MA
ATF6	Mouse		1:100	Abcam
ATF6	Rabbit	1:1000	1:200	Sigma‐Aldrich
XBP‐1	Rabbit	1:1000	1:200	Sigma‐Aldrich
GM130	Rabbit		1:150	Abcam
Phospho‐PERK	Rabbit	1:1000		Cell Signaling
Phospho‐Akt	Rabbit	1:2500		Cell Signaling
Akt	Rabbit	1:2500		Santa Cruz Biotechnology, Santa Cruz, CA
Phospho‐Erk1/2	Rabbit	1:2000		Cell Signaling
Erk2	Rabbit	1:1000		Santa Cruz Biotechnology
Secondary IgG				
Anti‐rabbit HRP	Goat	1:20000		BD Biosciences, San Jose, CA
Anti‐mouse HRP	Goat	1:20000		BD Biosciences
Anti‐rabbit CY3	Goat		1:1000	Jackson ImmunoResearch Laboratories, West Grove, PA
Anti‐mouse FITC	Goat		1:1000	Jackson ImmunoResearch Laboratories

WB, Western blot; IF, immunofluorescence, HRP, horseradish peroxidase.

### Animal studies

For dehydration experiments, C57B6 mice were divided into two groups, control animals with access to food and water ad libitum and animals deprived of water for 24 h. In some experiments, after microdissection, pools of 50 tubules were incubated for 3 h at 37°C in the presence or absence of 1 *μ*mol/L thapsigargin in medium (21 mmol/L Na‐gluconate, 1.2 mmol/L MgSO_4_, 2 mmol/L K_2_HPO_4_, 2 mmol/L Ca lactate, 1 mmol/L Na‐citrate, 5.5 mmol/L glucose, 12 mmol/L creatinine, 10 mmol/L HEPES and 5 mmol/L TRIS, pH 7.4) adjusted either at 300 mOsmol/kg or at 500 mOsmol/kg with NaCl. Cortical and medullary CD were microdissected from kidney slices as previously described (Roger et al. 1999). Each experimental group was constituted by pools of at least 50 tubules from the same animal. All animal experiments were approved by the Institutional Ethical Committee of Animal Care in Geneva and Cantonal authorities.

### Cell culture and transfection

mpkCCD_cl4_ (Bens et al. 1999) and mCCD_cl1_ (Gaeggeler et al. 2005) cells are spontaneously immortalized murine cell lines that express ENaC and display highly differentiated properties of CD principal cells (Bens et al. 1999; Gaeggeler et al. 2005). We previously used these cells to examine various hyperosmolality‐inducible events (Hasler et al. 2005, 2006, 2008a, b; Hasler 2009; Roth et al. 2010). Cells were cultured on permeable filters as previously described (Gaeggeler et al. 2005; Hasler et al. 2005) and transiently transfected using INTERFERin (Polyplus, Illikirch, France) with siRNA depicted in Table 2. All siRNA duplexes were made by Life Technologies (Carlsbad, CA). For hyperosmotic challenge, isosmotic medium (300 mOsmol/kg) was made hyperosmotic (350–500 mOsmol/kg) by substituting part of both apical (600 *μ*L total) and basal (1200 *μ*L total) medium with the appropriate amount of 1100 mOsmol/kg medium containing NaCl, urea or mannitol. Medium osmolality was checked using an osmometer (Advanced Instruments, Norwood, MA).

**Table 2.** PCR primer sequences (mouse unless specified)

Targeted gene	Forward	Reverse
Real‐Time PCR		
P_0_	AATCTCCAGAGGCACCATTG	GTTCAGCATGTTCAGCAGTG
P_0_ (rat)	CCTTCTCCTTCGGGCTGATC	GGGCTGTAGATGCTGCCATT
ENaC*α*	CAGACTTGGAGCTTTGACAAGGA	ACTTCTCTGTGCCTTGTTTATATGTGTT
ENaC*α* (rat)	CACTGTCTGCACCCTTAATCCTT	TGATGCGGTCCAGCTCTTC
ENaC*β*	CAGACTGGGCCTATTGCTATCTAAA	ACATGCTGAGGCAGGTCTCTCT
ENaC*β* (rat)	TGAGCAGGAAGGGTATTGTCAA	TTGTTGGCCGGCGATT
ENaC*γ*	CCGAGATCGAGACAGCAATGT	CGCTCAGCTTGAAGGATTCTG
ENaC*γ* (rat)	GATGGAGATCGAGACAGCAATG	CGCTCAGCTTGAAGGATTCTG
AQP2	CTTCCTTCGAGCTGCCTTC	CATTGTTGTGGAGAGCATTGAC
AQP2 (rat)	CGGTTGCTCCATGAATCCA	GAAGACCCAGTGATCATCAAACTTG
TNF*α*	GACCCTCACACTCAGATCATCTTCT	CCACTTGGTGGTTTGCTACGA
TonEBP	GCTTCAGCCCAAGGCATACA	GTCCCGGGCTGTGAGATG
AR	AGTGCGCATTGCTGAGAACTT	GTAGCTGAGTAGAGTGGCCATGTC
SGK1	CCAAACCCTCCGACTTTCAC	CCTTGTGCCTAGCCAGAAGAA
GILZ	GGTGGCCCTAGACAACAAGATT	GCGTACATCAGGTGGTTCTTCA
Grp94	TTGAACCTCTGCTCAACTGG	ATCCATACTGACTGGCCACA
Calreticulin	TCCGGTGTAAGGATGATGAA	AGTCCCAATCATCCTCCAAG
p58IPK	GTGGAGTAAATGCGGATGTG	TGCAGCGTGAAACTGTGATA
Grp78	AGGAGACTGCTGAGGCGTAT	CAGCATCTTTGGTTGCTTGT
ATF3	GAGGATTTTGCTAACCTGACACC	TTGACGGTAACTGACTCCAGC
EDEM1	TCGTCTTCGGCTATGACAAC	TTCAGATTGGAAGGGTCTCC
HERP	CACCTTGTGTGCAATGTGAA	ATTAGAGTTGTCCGGCTGCT
ATF6	TGAGCAGCTGAAGAAGGAGA	TTCTCTGACACCACCTCGTC
PERK	GGAAGGTCATGGCGTTTAGT	TTCTTCGCTGGCTGTGTAAC
IRE1	TTCGAGAATCAGACGAGCAC	CAAAGTCCTTCTGCTCCACA
XBP1	AAACAGAGTAGCAGCGCAGA	TCTTCCAAATCCACCACTTG
CHOP	GGAAACGGAAACAGAGTGGT	TCCTGCTCCTTCTCCTTCAT
PCR		
XBP1 (splice)	GAACCAGGAGTTAAGAACACG	AGGCAACAGTGTCAGAGTCC
siRNA		
Scramble	5′‐GCCACUCGUUUGUCGCCCUUGUAAA ‐3′	
PERK	5′‐CCAGAGGUGUUUGGGAACAAGAUGA ‐3′	
IRE1	5′‐CGGGCUCCAUCAAGUGGACUUUAAA ‐3′	
XBP‐1	5′‐ CAGCGCAGACUGCUCGAGAUAGAAA ‐3′	
ATF6	5′‐ CACAGACUCGUGUUCUUCAACUCAG ‐3′	

### RNA isolation and real‐time quantitative (Q) PCR

Total RNA from rat kidney sections, mouse microdissected CD and mpkCCD_cl4_ and mCCD_cl1_ cells was isolated using the NucleoSpin RNA II kit (Macherey‐Nagel, Düren, Germany) and reverse transcription and triplicate Q‐PCR amplification reactions were performed as previously described (Feraille et al. 2014). Primers used are depicted in Table 2. Ribosomal phosphoprotein P_0_ expression was used as a reliable internal standard. The fold change of test gene mRNA was expressed as 2^ΔΔCt^, where ΔCt is the difference in threshold cycles for the test gene and P_0_ and where ΔΔCt is the difference of ΔCt between stimulated (and/or cells transfected with siRNA) and non‐stimulated (and/or cells transfected with scramble siRNA) control. All primers were made by Microsynth (Balgach, Switzerland).

### PCR analysis of Xbp‐1 mRNA splicing

Total RNA from mCCD_cl1_ cells was isolated using the NucleoSpin RNA II kit (Macherey‐Nagel) and first strand cDNA was synthesized using SuperScript II Reverse Transcriptase (Life Technologies). Both spliced and unspliced Xbp‐1 mRNA were detected by PCR using primers depicted in Table 2. Experiments were repeated three times.

### Western blot analysis and preparation of nuclear and cytosolic extracts

Kidney tissue samples and mpkCCD_cl4_/mCCD_cl1_ cells were homogenized in 1 ml or 100 *μ*l, respectively, of lysis buffer and protein quantification, SDS‐PAGE and protein blotting were performed as previously described (Feraille et al. 2014). Antibody dilutions are depicted in Table 1. Horseradish peroxidase‐conjugated secondary antibodies (Table 1) were used for detection of immunoreactive proteins by chemiluminescence. Protein levels were quantified using ImageJ Java‐based image processing software (National Institutes of Health) after background subtraction. Test/loading control protein ratios were calculated and normalized to that of non‐stimulated (and/or cells transfected with scramble siRNA) control. Nuclear and cytosolic extracts were prepared as previously described (Roth et al. 2010) and 1 *μ*g of each extract was loaded on gels for Western blotting analysis.

### Fluorescence microscopy

Cells seeded on glass coverslips were fixed in 4% paraformaldehyde (PFA) for 20 min at room temperature and then mounted in SlowFade mounting medium (Life Technologies). Antibody dilutions are depicted in Table 1. For imaging of the endoplasmic reticulum (ER), cells were loaded with ER‐Tracker Red (Life Technologies), washed and fixed in PFA. Confocal imaging was performed on a Leica TCS SP5 MP confocal microscope system equipped with a 63X— 1.4 NA Plan‐apo HCS oil objective, using Leica Application Suite software (Leica Microsystems AG, Wetzlar, Germany). Confocal *z*‐stacks shown in Figures 4H, 5E and 5F are represented as maximum projections acquired using ImageJ.

### Statistics

Results are given as the mean ± SEM from *n* independent experiments. Each in vitro experiment was performed on cells from the same passage and all experiments were performed at least three times. The precise number of experiments performed is indicated in the Figure legends. All statistical analyses were performed using Prism software (Graphpad, La Jolla, CA). Significance between two pairs of experiments was determined using a student's *t*‐test.

## Results

### ENaC abundance in outer medullary CD is decreased by hyperosmolality

**Figure 1.**
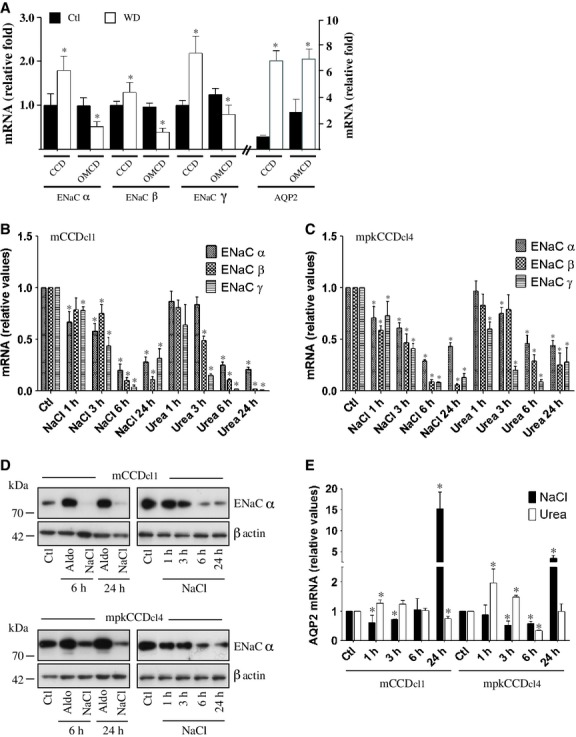
Hyperosmolality decreases ENaC abundance. (A) Q‐PCR analysis of ENaC and AQP2 mRNA abundance of microdissected CCD and OMCD from mice that were deprived of water for 24 h (WD) or given free access to water (Ctl). Data is represented as fold difference of expression over values obtained in CCD of Ctl homogenate and is expressed as the mean ± SEM of data from at least six animals. (B, C and E) mRNA abundance of ENaC (B and C) and AQP2 (E) in mCCD_cl1_ (B and E) and mpkCCD_cl4_ (C and E) cells challenged or not (Ctl) with NaCl or urea (500 mOsmol/kg) for 1–24 h. (D) Western blot against ENaC*α* from lysate of mCCD_cl1_ (top panel) and mpkCCD_cl4_ (bottom panel) cells challenged 6 or 24 h with aldosterone (1 *μ*mol/L) or 1–24 h with NaCl (500 mOsmol/kg). The blot against ENaC*α* at right was overexposed in order to better illustrate decreased ENaC*α* abundance by NaCl. *β*‐actin was used as a loading control. For (B), (C) and (E), data is represented as fold difference over non‐stimulated cells and is expressed as the mean ± SEM of four independent experiments.

Water deprivation increases plasma AVP concentration and consequently increases extracellular osmolality of the medulla but not cortex. We examined in vivo whether hyperosmolality affects ENaC mRNA expression by comparing cortical and medullary ENaC expression from microdissected CCD and OMCD of animals that were deprived of water for 24 h or given free access to water. We also compared changes of ENaC expression to those of AQP2. As previously reported (Kishore et al. 1996), AQP2 abundance in both CCD and OMCD was greatest in water‐deprived animals as compared to normally hydrated animals (Fig. 1A). Contrary to AQP2, dehydration decreased mRNA expression of all three ENaC subunits, but only in OMCD. In CCD, expression levels of all three ENaC subunits increased (Fig. 1A).

Water deprivation increases NaCl and, to a lesser extent, urea concentration in the outer medulla. This led us to examine the effects of NaCl and urea on ENaC mRNA abundance in vitro. Expression of all three ENaC subunits gradually decreased over time in both mCCD_cl1_ and mpkCCD_cl4_ cells challenged with NaCl (500 mOsmol/kg) or urea (500 mOsmol/kg), with a maximal effect observed after 6 h of challenge (Fig. 1B and C). Equiosmolar (500 mOsmol/kg) mannitol produced similar effects (not shown). NaCl also decreased ENaC*α* protein abundance in a time‐dependent manner (Fig. 1D). As we have previously shown (Hasler et al. 2005) NaCl, but not urea, first decreased but then increased AQP2 mRNA abundance (Fig. 1E). Collectively, our in vivo and in vitro data indicate that an increase of medullary environmental osmolality triggered by dehydration decreases ENaC abundance in OMCD.

### Hyperosmolality decreases ENaC abundance independently of aldosterone and TonEBP

**Figure 2.**
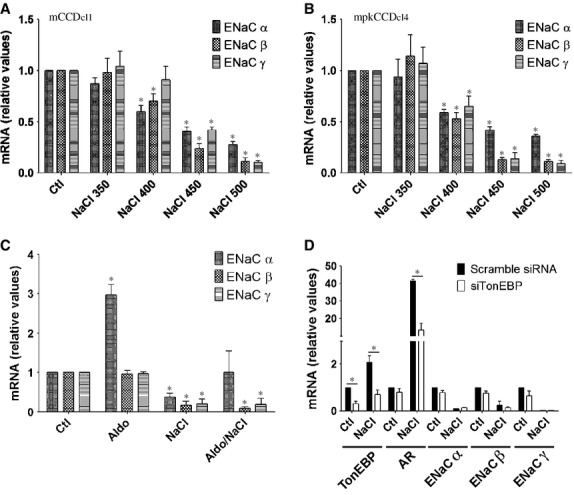
NaCl dose‐dependence and effects of aldosterone and TonEBP on decreased ENaC abundance by NaCl. (A and B) mRNA abundance of ENaC in mCCD_cl1_ (A) and mpkCCD_cl4_ (B) cells challenged or not (Ctl) 6 h with NaCl (350–500 mOsmol/kg). (C) mRNA abundance of ENaC in mCCD_cl1_ cells challenged or not (Ctl) with aldosterone (1 *μ*mol/L) or NaCl (500 mOsmol/kg) alone or with both stimuli for 6 h. Data is represented as fold difference over non‐stimulated cells and is expressed as the mean ± SEM of three independent experiments. (D) mCCD_cl1_ cells transfected with scramble siRNA or siRNA against TonEBP (siTonEBP) were challenged or not (Ctl) with NaCl for 6 h. TonEBP, aldose reductase (AR) and ENaC mRNA abundance was analyzed by Q‐PCR. Data is represented as fold difference over non‐stimulated cells transfected with scramble siRNA and is expressed as the mean ± SEM of three independent experiments.

Addition of NaCl to the cell medium decreased ENaC mRNA expression in a dose‐dependent manner in both mCCD_cl1_ (Fig. 2A) and mpkCCD_cl4_ (Fig. 2B) cells, with a maximal effect observed at 500 mOsmol/kg. We investigated the effects of aldosterone on decreased ENaC mRNA abundance by hyperosmolality. ENaC*α*, but not ENaC*β* or ENaC*γ*, was increased by aldosterone alone and NaCl decreased expression of each ENaC subunit in the absence or presence of aldosterone (Fig. 2C). This indicates that hyperosmolality decreases ENaC abundance independently of aldosterone.

TonEBP is a transcription factor whose activity is primarily regulated by extracellular tonicity (Jeon et al. 2006). We have previously shown that it may enhance *AQP2* gene transcription by NaCl (Hasler et al. 2006). siRNA targeting TonEBP significantly decreased NaCl‐enhanced mRNA expression of aldose reductase, a TonEBP target gene, but had no effect on mRNA expression of any ENaC subunit, either under isotonic or hypertonic conditions (Fig. 2D). These data indicate that hyperosmolality decreases ENaC abundance independently of TonEBP.

### Hyperosmotic stress induces an ER stress‐like response that decreases ENaC abundance via a transcriptionally mediated mechanism

**Figure 3.**
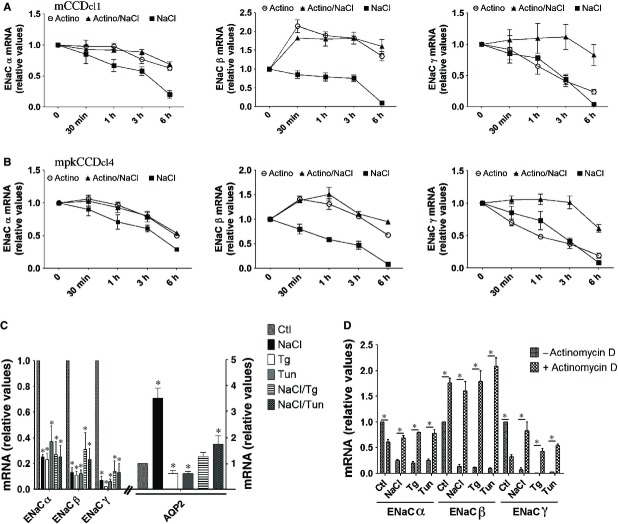
ER stress decreases ENaC abundance via a transcriptionally mediated mechanism. (A and B) mCCD_cl1_ (A) and mpkCCD_cl4_ (B) cells were challenged with either actinomycin D (5 *μ*mol/L) or NaCl (500 mOsmol/kg) alone or simultaneously challenged with both actinomycin D and NaCl for 30 min to 6 h. mRNA of all time points were extracted at the same time and ENaC abundance was analyzed by Q‐PCR. Data is represented as fold difference over values obtained in unstimulated cells (0 min) and is expressed as the mean ± SEM of four independent experiments. (C) mRNA abundance of ENaC and AQP2 in cells challenged or not (Ctl) with NaCl, thapsigargin (Tg, 1 *μ*mol/L), tunicamycin (Tun, 3 *μ*mol/L) or both NaCl and either chemical agent for 6 h. (D) mRNA abundance of ENaC in cells challenged or not with actinomycin D with or without (Ctl) NaCl, Tg or Tun for 6 h. Data is represented as fold difference over values obtained in unstimulated cells and is expressed as the mean ± SEM of four independent experiments.

We investigated ENaC mRNA stability under hyperosmotic conditions by treating mCCD_cl1_ (Fig. 3A) and mpkCCD_cl4_ (Fig. 3B) cells with actinomycin D, which halts de novo mRNA gene transcription. Cells were challenged with either actinomycin D or NaCl alone or were simultaneously challenged with both actinomycin D and NaCl for various periods of time (30 min to 6 h). Each experimental point was compared to the same control (untreated cells). Under control isosmotic conditions, actinomycin D decreased ENaC*α* and ENaC*γ* mRNA expression. Unexpectedly, actinomycin D increased expression of ENaC*β* mRNA. This implies the loss of short‐lived repressive element(s) that are quickly depleted in the presence of actinomycin D. Decreased ENaC mRNA abundance by hyperosmolality was blunted by actinomycin D, indicating that the effects of NaCl on ENaC abundance are transcriptionally mediated.

Decreased expression levels of all three ENaC subunits by hyperosmolality could be indicative of a single cellular event having global effects on gene expression. ER stress is a strong candidate for such an event. Both thapsigargin (Tg) and tunicamycin (Tun), two potent inducers of ER‐stress, decreased expression of all three ENaC subunits to extents that were similar, but not additive, to that achieved by NaCl (Fig. 3C). Tg and Tun decreased AQP2 abundance, but not to extents achieved for ENaC (Fig. 3C). Decreased ENaC abundance by either Tg or Tun was either blunted (ENaC*α* and ENaC*β*) or significantly reduced (ENaC*γ*) by actinomycin D (Fig. 3D). These data indicate that chemical induction of ER stress mimics the effects of hyperosmolality on ENaC expression.

**Figure 4.**
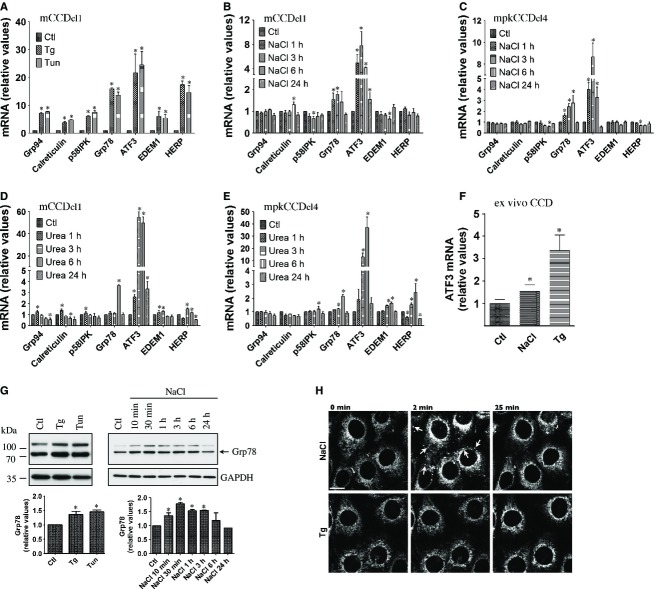
ER stress is transiently induced by NaCl. (A) mRNA abundance of ER‐stress responsive genes in mCCD_cl1_ cells challenged or not (Ctl) with thapsigargin (Tg, 1 *μ*mol/L) or tunicamycin (Tun, 3 *μ*mol/L) for 6 h. (B–E) mRNA abundance of ER‐stress responsive genes in mCCD_cl1_ (B and D) and mpkCCD_cl4_ (B) cells challenged or not with NaCl (500 mOsmol/kg, B and C) or urea (500 mOsmol/kg, D and E) for 1–24 h. Data is represented as fold difference over non‐stimulated cells and is expressed as the mean ± SEM of four independent experiments. (F) mRNA abundance of ATF3 in microdissected CCD challenged or not (Ctl) with NaCl (500 mOsmol/kg) or Tg (1 *μ*mol/L) for 3 h. Data is represented as fold difference over non‐stimulated CCD and is expressed as the mean ± SEM of data from at least six animals. (G) Western blot analysis of Grp78 protein in mCCD_cl1_ cells challenged or not (Ctl) with NaCl, Tg or Tun for 6 h (left panel) or NaCl for 10 min to 24 h (right panel). Data is represented as fold difference over non‐stimulated cells and is expressed as the mean ± SEM of three independent experiments. Representative Western blots are shown. GAPDH was used as a loading control. (H) Confocal maximum projections of mCCD_cl1_ cells loaded with ER‐Tracker Red challenged or not (0 min) with NaCl or Tg for 2 or 25 min. Shown are representative images of three similar experiments. Arrows depict the transient appearance of spherical clusters by NaCl. Bar, 10 *μ*mol/L.

We next examined whether hyperosmolality can induce ER stress and UPR signaling in vitro. ER stress activates the UPR, which in turn increases expression of genes that help restore ER homeostasis. We began our analysis of ER stress by hyperosmotic stress by examining the induction of genes that are typically upregulated by the UPR. We used Tg and Tun as control stimuli of ER stress. As expected, strong induction of ER stress by either Tg or Tun in mCCD_cl1_ cells dramatically increased UPR target gene expression (Fig. 4A). Some, but not all, of these genes were significantly upregulated by either NaCl (500 mOsmol/kg, Fig. 4B and C) or urea (500 mOsmol/kg, Fig. 4D and E) in both mCCD_cl1_ and mpkCCD_cl4_ cells. Of all genes tested, ATF3 mRNA upregulation by NaCl was strongest. Consistent with previous studies (Tian and Cohen 2002; Cai and Brooks 2011), this gene was also strongly upregulated in response to urea in both cell lines (Fig. 4D and E) and was also upregulated by either NaCl or Tg in microdissected CD (Fig. 4F). We continued our investigation of ER stress induction by hyperosmolality by examining Grp78 protein abundance (Fig. 4G). Similar to Grp78 mRNA, NaCl transiently increased Grp78 protein abundance. Induction peaked shortly following challenge, reaching levels similar to those achieved by either Tg or Tun (Fig. 4G). NaCl also altered ER morphology, as revealed by spherical clusters that immediately, but transiently, appeared upon NaCl but not Tg challenge (Fig. 4H). Possibly, an increase of intracellular osmolality upon NaCl challenge may affect ER volume, which could be connected with ER stress. Together, these data indicate that hyperosmolality transiently induces ER stress, albeit at levels lower than that achieved by chemical inducers.

### Hyperosmolality increases PERK and ATF6 activity

**Figure 5.**
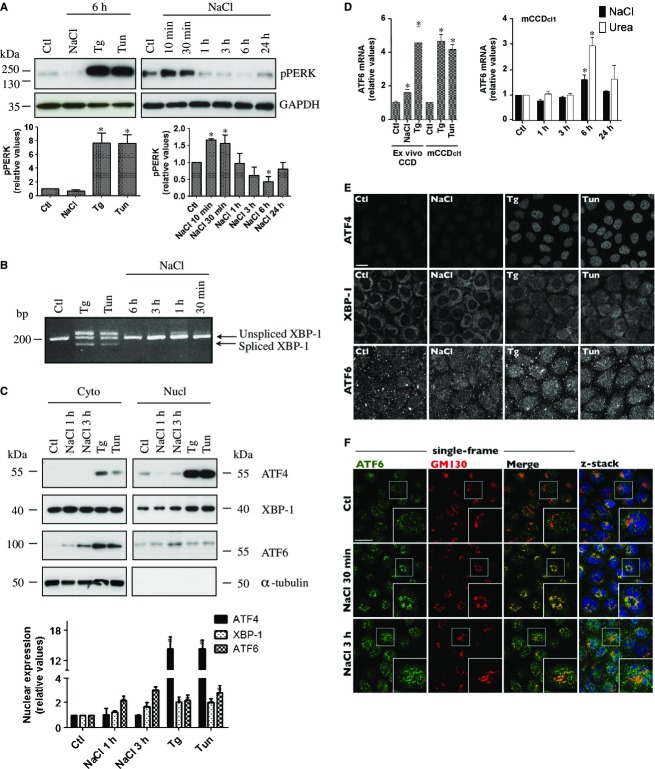
PERK and ATF6 activities are increased by NaCl. (A) Western blot analysis of phosphorylated PERK in mCCD_cl1_ cells challenged or not (Ctl) with NaCl (500 mOsmol/kg), thapsigargin (Tg, 1 *μ*mol/L) or tunicamycin (Tun, 3 *μ*mol/L) for 6 h (left panel) or NaCl for 10 min to 24 h (right panel). Data is represented as fold difference over non‐stimulated cells (Ctl) and is expressed as the mean ± SEM of three independent experiments. Representative Western blots are shown. GAPDH was used as a loading control. (B) PCR analysis of XBP‐1 mRNA splicing in lysates of cells challenged or not (Ctl) with Tg or Tun for 6 h or with NaCl for 30 min to 6 h. Bands >200 bp were consistently observed in samples from cells challenged with either chemical compound and may be experimental artifacts. A representative gel from three similar experiments is shown. (C) Western blot analysis of nuclear ATF4, XBP‐1 and ATF6 in cells challenged with NaCl for 1 or 3 h or Tg or Tun for 3 h. Data is represented as fold difference over non‐stimulated cells (Ctl) and is expressed as the mean ± SEM of three independent experiments. Representative Western blots of cytoplasmic and nuclear extracts are shown. *α*‐tubulin was used as a control of nuclear extract purity. (D) mRNA abundance of ATF6 in microdissected CCD challenged or not (Ctl) with NaCl (500 mOsmol/kg) or Tg (1 *μ*mol/L) for 3 h (left panel) or in mCCD_cl1_ cells challenged or not (Ctl) with Tg or Tun for 6 h (left panel) or with NaCl or urea (500 mOsmol/kg) for 1–24 h (right panel). Data is represented as fold difference over non‐stimulated CCD/cells and is expressed as the mean ± SEM of data from at least six animals or 4 experiments (for mCCD_cl1_ cells). (E) Confocal z‐stacks of ATF4, XBP‐1 and ATF6 in cells challenged or not (Ctl) with NaCl, Tg or Tun for 3 h. (F) Confocal single‐frame images and z‐stacks of ATF6 (green) and GM130 (red), a cis‐Golgi matrix protein, in cells challenged or not (Ctl) with NaCl for 30 min or 3 h. ATF6 accumulated in the Golgi after 30 min of challenge, as suggested by increased yellow staining, and in the nucleus (stained with Hoechst 33342, blue, shown in z‐stacks) after 3 h of challenge. Inserts depict enlarged images of regions depicted by white squares. For (E) and (F), representative images of three similar experiments are shown. Bar, 10 *μ*mol/L.

The mammalian UPR involves three canonical ER‐resident transmembrane proteins: RNA‐dependent protein kinase‐like ER kinase (PERK), inositol‐requiring ER‐to‐nucleus signal kinase 1 (IRE1*α*) and activating transcription factor 6 (ATF6). PERK phosphorylation, and consequent activation, was transiently increased by NaCl, albeit at lower levels than by either Tg or Tun, as would be expected (Fig. 5A). X box‐binding protein 1 (XBP1) mRNA splicing, used as an indicator of IRE1 activity, was induced by Tg and Tun but not NaCl (Fig. 5B). Analysis of nuclear ATF4, XBP‐1 and ATF6 expression, by Western blot analysis of nuclear extracts (Fig. 5C) and immunofluorescence (Fig. 5E) revealed that NaCl increased nuclear localization of XBP‐1 and ATF6 but not ATF4. ATF6 activation by NaCl was examined further. Tg and Tun increased, and NaCl transiently increased, ATF6 mRNA (Fig. 5D), reflecting increased ATF6 protein abundance (Fig. 5C). ATF6 mRNA levels were also significantly increased in microdissected CD challenged with either NaCl or Tg (Fig. 5D). We further investigated the ATF6 arm of the UPR by examining its intracellular distribution following NaCl challenge (Fig. 5F). ER stress increases translocation of an inactive precursor of ATF6 from the ER to the Golgi, where it is cleaved by Golgi‐resident proteases. The cytosolic fragment is an active transcription factor that translocates to the nucleus. Under isotonic conditions, low levels of ATF6 were observed in the nucleus and Golgi, as revealed by its co‐localization with Hoechst 33342, a nuclear stain, and GM130, a cis‐Golgi matrix protein (Fig. 5F). We have previously observed strong alterations of Golgi morphology upon NaCl challenge, characterized by the appearance of symmetrical Golgi structures that circumvent the centrosome (Nunes et al. 2013). These structural alterations were accompanied by an accumulation of ATF6 in the Golgi after 30 min of NaCl challenge (Fig. 5F), indicating increased translocation of pre‐existing ATF6 to the Golgi. Golgi morphology recovered after 3 h of NaCl challenge, at which time ATF6 accumulated in both the Golgi and nucleus (Fig. 5F), presumably as a consequence of de novo ATF6 protein synthesis (Fig. 5C and D). ATF6 signal specificity was verified using another anti‐ATF6 antibody (Sigma‐Aldrich) and the signal obtained by either antibody was abated by siRNA against ATF6 (not shown). Together, these observations indicate that while Tg and Tun strongly activate PERK, IRE1*α* and ATF6, NaCl activates PERK and ATF6.

### Increased PERK and ATF6 activity upon hyperosmotic stress differently affect each ENaC subunit

**Figure 6.**
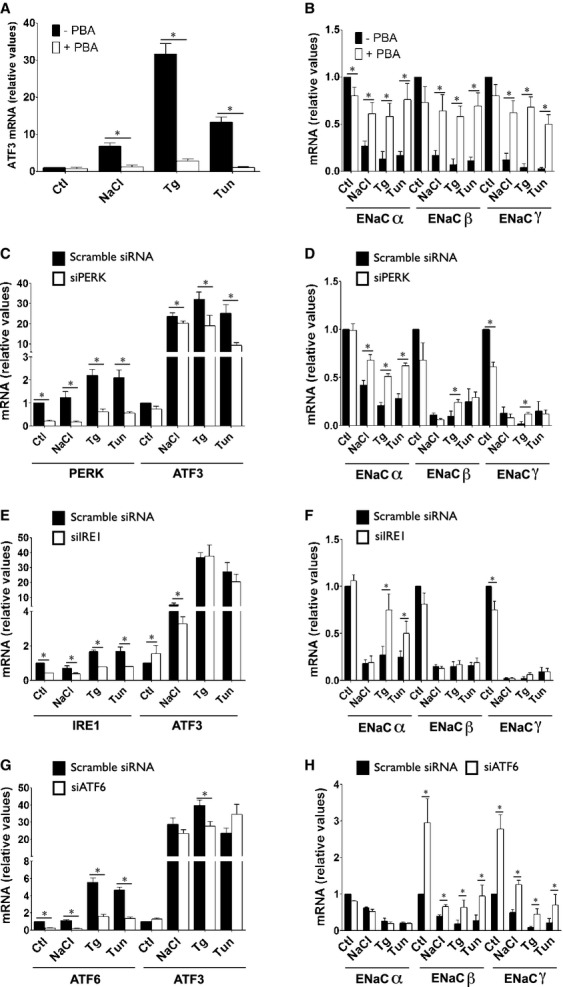
PERK, IRE*α* and ATF6 differently influence abundance of each ENaC subunit. (A and B) mRNA abundance of ATF3 (A) and ENaC (B) in cells challenged or not with NaCl (500 mOsmol/kg), Tg (Tg, 1 *μ*mol/L) or Tun (Tun, 3 *μ*mol/L) in the presence or absence (Ctl) of 4‐phenylbutyric acid (PBA, 20 mmol/L) for 6 h. Data is represented as fold difference over non‐stimulated cells and is expressed as the mean ± SEM of four independent experiments. (C–H) Cells transfected with scramble siRNA or siRNA against PERK (siPERK, C and D), IRE1 (siIRE1, E and F) or ATF6 (siATF6, G and H) were challenged or not (Ctl) with NaCl, Tg or Tun for 6 h. Target gene and ATF3 (C, E and G) and ENaC (D, F and H) mRNA abundance was analyzed by Q‐PCR. Data is represented as fold difference over non‐stimulated cells transfected with scramble siRNA and is expressed as the mean ± SEM of four independent experiments.

We examined the effects of modulated UPR activity on ENaC abundance. Chemical attenuation of ER stress by 4‐phenylbutyric acid (PBA) abolished induction of ER‐stress responsive genes by NaCl, Tg and Tun, as illustrated by blunted ATF3 induction (Fig. 6A). PBA also abolished the effects of NaCl, Tg and Tun on all three ENaC subunits (Fig. 6B). We next investigated how each UPR arm affects ENaC abundance by transfecting cells with siRNA against PERK, IRE1*α* or ATF6. Globally, each siRNA reduced ATF3 induction by NaCl, Tg or Tun but to a significantly smaller extent than PBA (Fig. 6C, E and G). This may be expected since decreased activity of any one UPR mediator is likely to be at least partly compensated by other UPR arms (Ron and Walter 2007). Because simultaneous downregulation of all three UPR mediators blunted cell proliferation, we were unable to analyze their combined effects. Attenuated PERK expression partly blunted the effect of NaCl, Tg and Tun on ENaC*α*, but not ENaC*β* or ENaC*γ* (Fig. 6D). Similar results were obtained when IRE1*α* expression was attenuated, but only in cells challenged with Tg or Tun (Fig. 6F), corroborating our observation that IRE1*α* is not significantly activated by NaCl (Fig. 5B). XBP‐1 silencing by siRNA did not significantly affect decreased abundance of any ENaC subunit by either NaCl, Tg or Tun (not shown). Attenuated ATF6 expression had no effect on ENaC*α* abundance but increased basal levels of both ENaC*β* and ENaC*γ* mRNA and partly blunted their decreased abundance by NaCl, Tg and Tun (Fig. 6H).

## Discussion

**Figure 7.**
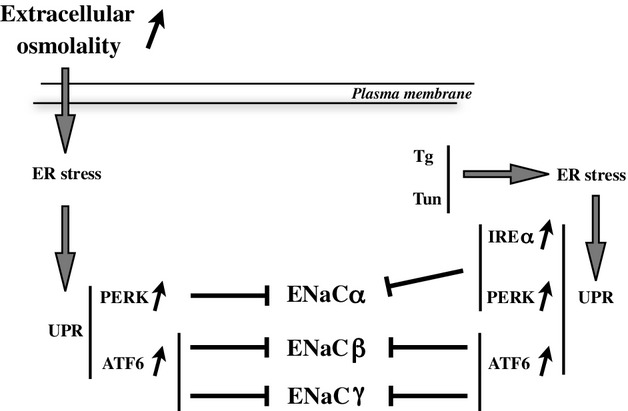
Proposed model of decreased ENaC abundance by hyperosmolality. Increased extracellular osmolality induces transient ER stress and activation of the PERK and ATF6 arms of the UPR. Through a transcriptionally‐mediated mechanism, PERK signaling down‐regulates ENaC*α* while ATF6 signaling decreases ENaC*β* and ENaC*γ* expression. The effects of PERK and ATF6 signaling on each ENaC subunit were reproduced by thapsigargin (Tg) and tunicamycin (Tun). Although IRE1*α* activation by hyperosmolality was not detected, chemical induction of its activity attenuated ENaC*α* expression.

Interference of ER protein folding and membrane trafficking leads to ER stress (Rutkowski and Kaufman 2004; Ron and Walter 2007). Hyperosmolality affects cellular homeostasis and structural organization (Carpentier et al. 1989; Heuser and Anderson 1989; Ellis and Minton 2006; Choe and Strange 2008; Hasler et al. 2008b; Munishkina et al. 2008; Zhou et al. 2008; Burkewitz et al. 2011; Nunes et al. 2013) and previous observations (Kultz et al. 1998; Zhang et al. 1999; Tian and Cohen 2002; van Balkom et al. 2004; Hoorn et al. 2005; Cai et al. 2006, 2010; Dihazi et al. 2011) strongly suggest that hypertonicity induces ER stress in the renal medulla, consistent with our data. In the present study, we examined how hypertonicity affects the UPR in CD principal cells and how this affects ENaC abundance. As summarized in Figure 7, hypertonicity was found to increase activity of the PERK and ATF6 arms of the UPR. Our data indicate that ENaC*α* abundance is downregulated by PERK signaling while ENaC*β* and ENaC*γ* abundance in non‐stimulated cells is maintained at low levels by basal ATF6 activity, whose activation by NaCl further decreases abundance of both ENaC subunits.

Our study contributes to the literature that show that hyperosmotic stress induces an ER stress‐like response. Water restriction or dDAVP infusion, which both increase medullary osmolality, increased Grp78 and ATF4 expression in the inner medulla (van Balkom et al. 2004; Cai et al. 2006, 2010). Conversely, GRP78 protein abundance decreased in IMCD of animals subjected to vasopressin escape (Hoorn et al. 2005). Expression levels of ER stress‐responsive genes CHOP and ATF3 were increased by either NaCl or urea challenge in cultured IMCD cells (Kultz et al. 1998; Zhang et al. 1999; Tian and Cohen 2002) and several ER stress‐responsive genes were upregulated by NaCl challenge in renal fibroblast cell lines (Dihazi et al. 2011). Our study shows that overall, the extent of induction of ER stress‐responsive genes by either Tg or Tun was significantly higher than that induced by hyperosmolality. However, it should be noted that induction of ER stress by either chemical agent would be expected to be much higher than that induced by a more physiological stimulus since the effects of both chemical agents accumulate over time and eventually induce apoptosis. In this respect, the situation for hyperosmotic stress is very different. Indeed, cells recover from osmotic stress and hyperosmolality may be expected to only transiently increase ER stress‐responsive gene expression, as was observed. The observation that ER stress‐responsive genes were similarly induced by NaCl and urea indicates that these effects are induced by an increase of intracellular osmolality, and not by NaCl *per se*.

To the best of our knowledge, very little is known of its effects on UPR signaling. Bioinformatic pathway analysis of proteins regulated by vasopressin escape indicates that ATF6, XBP1 and eIF2*α* are associated with this response (Hoorn et al. 2005). NaCl (1 h) was shown to increase eIF2*α* phosphorylation in mouse reticulocytes (Lu et al. 2001). Another study found that urea increased eIF2*α* phosphorylation, ATF3 mRNA and protein abundance in cultured IMCD cells (1–6 h), although the authors attributed eIF2*α* kinase phosphorylation to general control kinase GCN2 rather than PERK (Cai and Brooks 2011). Our study suggests that increased UPR activity by NaCl is primarily associated with activation of PERK and ATF6 but not IRE1*α* in cultured CD principal cells. IRE1*α* activity was previously found to be inhibited when bound to an aberrant splicing isoform of presenilin‐2 caused by hypoxia or oxidative stress (Katayama et al. 2004). Possibly, a similar mechanism may occur in osmotically‐challenged cells. Upregulated expression of only relatively few genes by ER stress can be tied definitively to a specific canonical UPR transducer (Arensdorf et al. 2013). Our data indicate that unlike chemical chaperones that blunted hyperosmolality‐induced ENaC downregulation, siRNA against canonical ER stress transducers only partly reduced this effect. This can likely be accounted for by crosstalk between canonical UPR arms and/or activation of non‐canonical UPR pathways (Arensdorf et al. 2013) by hyperosmolality. For instance, nuclear accumulation of factors like bZIPs and C/EBP*α*,*β* by PERK (Arensdorf et al. 2013) might occur in osmotically challenged cells independently of the PERK canonical downstream target ATF4. Although IRE1*α* activation by hyperosmolality might be too mild or transient to be detected by our assays, this may also help explain how XBP‐1 translocates to the nucleus upon hypertonic challenge independently of an increase of IRE1*α* activity. On the other hand, manipulation of IRE1*α* affected down‐regulated ENaC*α* expression by chemical ER stress induction, suggesting that other sources of ER stress that induce the IRE1*α* pathway may affect ENaC*α* expression.

Salt and water reabsorption by the CD is far better understood than coupling between both processes. Water flow across CD principal cells relies on apical AQP2 expression and apical ENaC constitutes a rate‐limiting step for Na^+^ transport across the apical membrane of these cells. Several pieces of experimental data indicate that Na^+^ transport across cortical CD epithelia is isosmotically coupled to water transport (Kudo et al. 1994; Nicco et al. 2001; Hasler et al. 2003; Bugaj et al. 2009; Gaeggeler et al. 2011). This is likely not the case for medullary CD since the abundance of ENaC, but not AQP2, decreases along the CD, being highest in the CCD and lowest in the IMCD (Duc et al. 1994; Vehaskari et al. 1998; MacDonald et al. 2000; Kim et al. 2004; Frindt et al. 2007). Our study suggests a role for hyperosmolality in uncoupling water flow from Na^+^ transport. We illustrate this principle by water deprivation, which increases outer medulla osmolality by several hundred mOsmol/kg. Under these conditions, reabsorption of isosmotic fluid would help restore blood volume but extracellular osmolality would remain elevated. Increasing free water reabsorption, via increased AQP2 and decreased ENaC expression, would help resolve this dilemma. Such uncoupling would endow the OMCD with the ability to recalibrate water and salt transport and help correct electrolyte imbalance.

Medullary, but not cortical, osmolality varies with circulating AVP levels. Dehydration increases plasma AVP concentration. Under these conditions, AQP2 abundance is increased in both the CCD and OMCD (our study and (Kishore et al. 1996)). On the other hand, our study shows that mRNA expression of all three ENaC subunits increased in the CCD but decreased in the OMCD. Consistent with this study, ENaC*β* and ENaC*γ* mRNA expression was increased in the renal cortex of Sprague Dawley rats after 5 days of moderate water restriction (Nicco et al. 2001). AVP increases Na^+^ transport by CCD (Tomita et al. 1985; Reif et al. 1986; Hawk et al. 1996; Djelidi et al. 1997; Nicco et al. 2001; Bugaj et al. 2009) and increases ENaC*β* and ENaC*γ*, but not ENaC*α*, abundance in a rat CCD cell line (RCCD_1_) (Djelidi et al. 1997). ENaC*β* and ENaC*γ* mRNA were also increased by dDAVP infusion (5 days) in the renal cortex of Sprague Dawley rats and Brattleboro rats lacking endogenous AVP (Nicco et al. 2001). In another study, protein levels of all three ENaC subunits were increased in the renal cortex of Brattleboro rats by acute (≤6 h) or prolonged (7 days) dDAVP infusion (Ecelbarger et al. 2000). Consequently, dehydration may increase ENaC abundance in the CCD via a direct effect of AVP. On the other hand, despite increased plasma AVP concentration, ENaC was downregulated in the OMCD of dehydrated animals. This is in line with a previous study (Ecelbarger et al. 2000), which showed increased protein expression of all three ENaC subunits in cortex, but not medulla, of Brattleboro rats acutely (≤1 h) administered with dDAVP. Under conditions of dehydration, increased ENaC*β* and ENaC*γ* abundance by AVP in the CCD may be complemented, when dehydration is severe, by an increase of ENaC*α* abundance by aldosterone following activation of the renin‐angiotensin aldosterone system. Our data show that hyperosmolality decreases upregulated ENaC*α* abundance by aldosterone in vitro, indicating that the effect of hyperosmolality in the outer medulla may prevail both in the absence and presence of aldosterone in vivo.

ENaC protein is inefficiently assembled in the ER and a significant percentage of each subunit is targeted for ER‐associated degradation (Staub et al. 1997; Buck et al. 2013). The present study revealed decreased expression of all three ENaC subunits by ER stress, induced by either chemicals or hyperosmolality. Our data indicate that the UPR is involved in this event. Interestingly, while most attention has focused on transcriptional upregulation of genes by the UPR, up to half of all genes regulated by ER stress are actually suppressed (Arensdorf and Rutkowski 2013; Arensdorf et al. 2013). It is also becoming increasingly clear that the UPR is deeply rooted in cellular physiology and regulates genes that do not seemingly affect ER function (Arensdorf and Rutkowski 2013; Arensdorf et al. 2013). As part of a protective response, increased intracellular Na^+^ concentration decreases both ENaC open probability and expression at the cell surface (Kashlan and Kleyman 2012), thereby averting excessive Na^+^ entry into cells. This may hold particularly true for renal medullary cells of dehydrated animals since an efficient urinary concentrating mechanism is accompanied by hypoxia (Brezis et al. 1989). It is therefore conceivable that hyperosmotic activation of the UPR might contribute to the protective response by decreasing ENaC abundance. Our data reveal that hyperosmolality does not immediately decrease ENaC abundance. Instead, it gradually decreases over time following challenge. This indicates that the transient increase of UPR signaling by hyperosmolality is sufficient to induce de novo gene transcription, as corroborated by our pharmacological data. This may involve upregulated activity of complexes that repress *ENaC* gene transcription, like those reported to repress ENaC*α* gene transcription (Zentner et al. 2001; Zhang et al. 2006, 2006, 2007). Alternatively, the UPR may also decrease transcription of factors that degrade ENaC mRNA. Increased abundance of ENaC*β* mRNA by actinomycin D point to this possibility, at least for this subunit. In this respect, modulation of ENaC abundance by microRNA might be particularly pertinent, as illustrated by the effects of hyperosmolality on the cellular abundance of numerous miR (Huang et al. 2011).

In conclusion, our study provides strong evidence that hyperosmolality attenuates ENaC abundance. This at least partly relies on transient activation of PERK and ATF6. The effects of hyperosmolality are partly abrogated by chemical attenuation of ER stress, depleted expression of UPR transducers and inhibition of transcriptional activity. These observations reveal a role for the UPR in uncoupled AQP2 and ENaC abundance that would allow the OMCD to re‐equilibrate water and salt excretion under conditions of electrolyte imbalance.

## Conflict of Interest

No conflicts of interest, financial or otherwise, are declared by the authors.

## References

[b1] Arensdorf A. M. Rutkowski D. T. 2013 Endoplasmic reticulum stress impairs IL‐4/IL‐13 signaling through C/EBPbeta‐mediated transcriptional suppression. J. Cell Sci.; 126:4026-4036. 23813955 10.1242/jcs.130757 PMC3757336

[b2] Arensdorf A. M. Diedrichs D. Rutkowski D. T. 2013 Regulation of the transcriptome by ER stress: non‐canonical mechanisms and physiological consequences. Front. Genet.; 4:256 24348511 10.3389/fgene.2013.00256 PMC3844873

[b3] van Balkom B. W. Hoffert J. D. Chou C. L. Knepper M. A. 2004 Proteomic analysis of long‐term vasopressin action in the inner medullary collecting duct of the Brattleboro rat. Am. J. Physiol. Renal. Physiol.; 286:F216-F224. 14532164 10.1152/ajprenal.00307.2003

[b4] Bankir L. Fernandes S. Bardoux P. Bouby N. Bichet D. G. 2005 Vasopressin‐V2 receptor stimulation reduces sodium excretion in healthy humans. J. Am. Soc. Nephrol.; 16:1920-1928. 15888562 10.1681/ASN.2004121079

[b5] Bens M. Vallet V. Cluzeaud F. Pascual‐Letallec L. Kahn A. Rafestin‐Oblin M. E. 1999 Corticosteroid‐dependent sodium transport in a novel immortalized mouse collecting duct principal cell line. J. Am. Soc. Nephrol.; 10:923-934. 10232677 10.1681/ASN.V105923

[b6] Brezis M. Rosen S. N. Epstein F. H. 1989 The pathophysiological implications of medullary hypoxia. Am. J. Kidney Dis.; 13:253-258. 2493191 10.1016/s0272-6386(89)80062-9

[b7] Buck T. M. Plavchak L. Roy A. Donnelly B. F. Kashlan O. B. Kleyman T. R. 2013 The Lhs1/GRP170 chaperones facilitate the endoplasmic reticulum‐associated degradation of the epithelial sodium channel. J. Biol. Chem.; 288:18366-18380. 23645669 10.1074/jbc.M113.469882 PMC3689978

[b8] Bugaj V. Pochynyuk O. Stockand J. D. 2009 Activation of the epithelial Na+ channel in the collecting duct by vasopressin contributes to water reabsorption. Am. J. Physiol. Renal. Physiol.; 297:F1411-F1418. 19692483 10.1152/ajprenal.00371.2009 PMC2781343

[b9] Burkewitz K. Choe K. Strange K. 2011 Hypertonic stress induces rapid and widespread protein damage in C. elegans. Am. J. Physiol. Cell Physiol.; 301:C566-C576. 21613604 10.1152/ajpcell.00030.2011 PMC3174568

[b10] Cai Q. Brooks H. L. 2011 Phosphorylation of eIF2alpha via the general control kinase, GCN2, modulates the ability of renal medullary cells to survive high urea stress. Am. J. Physiol. Renal. Physiol.; 301:F1202-F1207. 21880833 10.1152/ajprenal.00272.2011 PMC3233868

[b11] Cai Q. Keck M. McReynolds M. R. Klein J. D. Greer K. Sharma K. 2006 Effects of water restriction on gene expression in mouse renal medulla: identification of 3betaHSD4 as a collecting duct protein. Am. J. Physiol. Renal. Physiol.; 291:F218-F224. 16478974 10.1152/ajprenal.00413.2005

[b12] Cai Q. Nelson S. K. McReynolds M. R. Diamond‐Stanic M. K. Elliott D. Brooks H. L. 2010 Vasopressin increases expression of UT‐A1, UT‐A3, and ER chaperone GRP78 in the renal medulla of mice with a urinary concentrating defect. Am. J. Physiol. Renal. Physiol.; 299:F712-F719. 20668095 10.1152/ajprenal.00690.2009 PMC2957250

[b13] Carpentier J. L. Sawano F. Geiger D. Gorden P. Perrelet A. Orci L. 1989 Potassium depletion and hypertonic medium reduce “non‐coated” and clathrin‐coated pit formation, as well as endocytosis through these two gates. J. Cell. Physiol.; 138:519-526. 2466853 10.1002/jcp.1041380311

[b14] Choe K. P. Strange K. 2008 Genome‐wide RNAi screen and in vivo protein aggregation reporters identify degradation of damaged proteins as an essential hypertonic stress response. Am. J. Physiol. Cell Physiol.; 295:C1488-C1498. 18829898 10.1152/ajpcell.00450.2008 PMC2603564

[b15] Dihazi H. Dihazi G. H. Mueller C. Lahrichi L. Asif A. R. Bibi A. 2011 Proteomics characterization of cell model with renal fibrosis phenotype: osmotic stress as fibrosis triggering factor. J. Proteomics; 74:304-318. 21118732 10.1016/j.jprot.2010.11.007

[b16] Djelidi S. Fay M. Cluzeaud F. Escoubet B. Eugene E. Capurro C. 1997 Transcriptional regulation of sodium transport by vasopressin in renal cells. J. Biol. Chem.; 272:32919-32924. 9407070 10.1074/jbc.272.52.32919

[b17] Duc C. Farman N. Canessa C. M. Bonvalet J. P. Rossier B. C. 1994 Cell‐specific expression of epithelial sodium channel alpha, beta, and gamma subunits in aldosterone‐responsive epithelia from the rat: localization by in situ hybridization and immunocytochemistry. J. Cell Biol.; 127:1907-1921. 7806569 10.1083/jcb.127.6.1907 PMC2120291

[b18] Ecelbarger C. A. Kim G. H. Terris J. Masilamani S. Mitchell C. Reyes I. 2000 Vasopressin‐mediated regulation of epithelial sodium channel abundance in rat kidney. Am. J. Physiol. Renal. Physiol.; 279:F46-F53. 10894786 10.1152/ajprenal.2000.279.1.F46

[b19] Ellis R. J. Minton A. P. 2006 Protein aggregation in crowded environments. Biol. Chem.; 387:485-497. 16740119 10.1515/BC.2006.064

[b20] Feraille E. Dizin E. Roth I. Derouette J. P. Szanto I. Martin P. Y. 2014 NADPH oxidase 4 deficiency reduces aquaporin‐2 mRNA expression in cultured renal collecting duct principal cells via increased PDE3 and PDE4 activity. PLoS ONE; 9:e87239 24466344 10.1371/journal.pone.0087239 PMC3900718

[b21] Frindt G. Ergonul Z. Palmer L. G. 2007 Na channel expression and activity in the medullary collecting duct of rat kidney. Am. J. Physiol. Renal. Physiol.; 292:F1190-F1196. 17200158 10.1152/ajprenal.00399.2006

[b22] Fushimi K. Uchida S. Hara Y. Hirata Y. Marumo F. Sasaki S. 1993 Cloning and expression of apical membrane water channel of rat kidney collecting tubule. Nature; 361:549-552. 8429910 10.1038/361549a0

[b23] Gaeggeler H. P. Gonzalez‐Rodriguez E. Jaeger N. F. Loffing‐Cueni D. Norregaard R. Loffing J. 2005 Mineralocorticoid versus glucocorticoid receptor occupancy mediating aldosterone‐stimulated sodium transport in a novel renal cell line. J. Am. Soc. Nephrol.; 16:878-891. 15743993 10.1681/ASN.2004121110

[b24] Gaeggeler H. P. Guillod Y. Loffing‐Cueni D. Loffing J. Rossier B. C. 2011 Vasopressin‐dependent coupling between sodium transport and water flow in a mouse cortical collecting duct cell line. Kidney Int.; 79:843-852. 21178974 10.1038/ki.2010.486

[b25] Hasler U. 2009 Controlled aquaporin‐2 expression in the hypertonic environment. Am. J. Physiol. Cell Physiol.; 296:C641-C653. 19211910 10.1152/ajpcell.00655.2008

[b26] Hasler U. Mordasini D. Bianchi M. Vandewalle A. Feraille E. Martin P.‐Y. 2003 Dual influence of aldosterone on AQP2 expression in cultured renal collecting duct principal cells. J. Biol. Chem.; 278:21639-21648. 12660245 10.1074/jbc.M212388200

[b27] Hasler U. Vinciguerra M. Vandewalle A. Martin P.‐Y. Feraille E. 2005 Dual effects of hypertonicity on aquaporin‐2 expression in cultured renal collecting duct principal cells. J. Am. Soc. Nephrol.; 16:1571-1582. 15843469 10.1681/ASN.2004110930

[b28] Hasler U. Jeon U. S. Kim J. A. Mordasini D. Kwon H. M. Feraille E. 2006 Tonicity‐responsive enhancer binding protein is an essential regulator of aquaporin‐2 expression in renal collecting duct principal cells. J. Am. Soc. Nephrol.; 17:1521-1531. 16641150 10.1681/ASN.2005121317

[b29] Hasler U. Leroy V. Jeon U. S. Bouley R. Dimitrov M. Kim J. A. 2008a NF‐kappa B modulates aquaporin‐2 transcription in renal collecting duct principal cells. J. Biol. Chem.; 283:28095-28105. 18703515 10.1074/jbc.M708350200 PMC2568939

[b30] Hasler U. Nunes P. Bouley R. Lu H. A. Matsuzaki T. Brown D. 2008b Acute hypertonicity alters aquaporin‐2 trafficking and induces a MAP kinase‐dependent accumulation at the plasma membrane of renal epithelial cells. J. Biol. Chem.; 283:26643-26661. 18664568 10.1074/jbc.M801071200 PMC2546545

[b31] Hawk C. T. Li L. Schafer J. A. 1996 AVP and aldosterone at physiological concentrations have synergistic effects on Na+ transport in rat CCD. Kidney Int. Suppl.; 57:S35-S41. 8941920

[b32] Heuser J. E. Anderson R. G. 1989 Hypertonic media inhibit receptor‐mediated endocytosis by blocking clathrin‐coated pit formation. J. Cell Biol.; 108:389-400. 2563728 10.1083/jcb.108.2.389 PMC2115439

[b33] Hoorn E. J. Hoffert J. D. Knepper M. A. 2005 Combined proteomics and pathways analysis of collecting duct reveals a protein regulatory network activated in vasopressin escape. J. Am. Soc. Nephrol.; 16:2852-2863. 16079266 10.1681/ASN.2005030322 PMC1400600

[b34] Huang W. Liu H. Wang T. Zhang T. Kuang J. Luo Y. 2011 Tonicity‐responsive microRNAs contribute to the maximal induction of osmoregulatory transcription factor OREBP in response to high‐NaCl hypertonicity. Nucleic Acids Res.; 39:475-485. 20852262 10.1093/nar/gkq818 PMC3025551

[b35] Jeon U. S. Kim J. A. Sheen M. R. Kwon H. M. 2006 How tonicity regulates genes: story of TonEBP transcriptional activator. Acta Physiol. (Oxf); 187:241-247. 16734761 10.1111/j.1748-1716.2006.01551.x

[b36] Kashlan O. B. Kleyman T. R. 2012 Epithelial Na(+) channel regulation by cytoplasmic and extracellular factors. Exp. Cell Res.; 318:1011-1019. 22405998 10.1016/j.yexcr.2012.02.024 PMC3698959

[b37] Katayama T. Imaizumi K. Manabe T. Hitomi J. Kudo T. Tohyama M. 2004 Induction of neuronal death by ER stress in Alzheimer's disease. J. Chem. Neuroanat.; 28:67-78. 15363492 10.1016/j.jchemneu.2003.12.004

[b38] Kim S. W. Wang W. Nielsen J. Praetorius J. Kwon T. H. Knepper M. A. 2004 Increased expression and apical targeting of renal ENaC subunits in puromycin aminonucleoside‐induced nephrotic syndrome in rats. Am. J. Physiol. Renal. Physiol.; 286:F922-F935. 15075188 10.1152/ajprenal.00277.2003

[b39] Kishore B. K. Terris J. M. Knepper M. A. 1996 Quantitation of aquaporin‐2 abundance in microdissected collecting ducts: axial distribution and control by AVP. Am. J. Physiol.; 271:F62-F70. 8760244 10.1152/ajprenal.1996.271.1.F62

[b40] Kudo L. H. Hawk C. T. Schafer J. A. 1994 Sodium and water transport in cortical collecting duct of Dahl salt‐resistant rat. Am. J. Physiol.; 267:F583-F591. 7943356 10.1152/ajprenal.1994.267.4.F583

[b41] Kultz D. Madhany S. Burg M. B. 1998 Hyperosmolality causes growth arrest of murine kidney cells. Induction of GADD45 and GADD153 by osmosensing via stress‐activated protein kinase 2. J. Biol. Chem.; 273:13645-13651. 9593703 10.1074/jbc.273.22.13645

[b42] Lu L. Han A. P. Chen J. J. 2001 Translation initiation control by heme‐regulated eukaryotic initiation factor 2alpha kinase in erythroid cells under cytoplasmic stresses. Mol. Cell. Biol.; 21:7971-7980. 11689689 10.1128/MCB.21.23.7971-7980.2001 PMC99965

[b43] MacDonald P. MacKenzie S. Ramage L. E. Seckl J. R. Brown R. W. 2000 Corticosteroid regulation of amiloride‐sensitive sodium‐channel subunit mRNA expression in mouse kidney. J. Endocrinol.; 165:25-37. 10750033 10.1677/joe.0.1650025

[b44] Morris R. G. Uchida S. Brooks H. Knepper M. A. Chou C. L. 2005 Altered expression profile of transporters in the inner medullary collecting duct of aquaporin‐1 knockout mice. Am. J. Physiol. Renal. Physiol.; 289:F194-F199. 15713911 10.1152/ajprenal.00121.2004

[b45] Munishkina L. A. Ahmad A. Fink A. L. Uversky V. N. 2008 Guiding protein aggregation with macromolecular crowding. Biochemistry; 47:8993-9006. 18665616 10.1021/bi8008399 PMC2676887

[b46] Nicco C. Wittner M. DiStefano A. Jounier S. Bankir L. Bouby N. 2001 Chronic exposure to vasopressin upregulates ENaC and sodium transport in the rat renal collecting duct and lung. Hypertension; 38:1143-1149. 11711512 10.1161/hy1001.092641

[b47] Nielsen S. DiGiovanni S. R. Christensen E. I. Knepper M. A. Harris H. W. 1993 Cellular and subcellular immunolocalization of vasopressin‐regulated water channel in rat kidney. Proc. Natl Acad. Sci. USA; 90:11663-11667. 8265605 10.1073/pnas.90.24.11663 PMC48044

[b48] Nielsen J. Kwon T. H. Praetorius J. Frokiaer J. Knepper M. A. Nielsen S. 2006 Aldosterone increases urine production and decreases apical AQP2 expression in rats with diabetes insipidus. Am. J. Physiol. Renal. Physiol.; 290:F438-F449. 16159898 10.1152/ajprenal.00158.2005

[b49] Nunes P. Ernandez T. Roth I. Qiao X. Strebel D. Bouley R. 2013 Hypertonic stress promotes autophagy and microtubule‐dependent autophagosomal clusters. Autophagy; 9:550-567. 23380587 10.4161/auto.23662 PMC3627670

[b50] Picard N. Eladari D. El Moghrabi S. Planes C. Bourgeois S. Houillier P. 2008 Defective ENaC processing and function in tissue kallikrein‐deficient mice. J. Biol. Chem.; 283:4602-4611. 18086683 10.1074/jbc.M705664200

[b51] Reif M. C. Troutman S. L. Schafer J. A. 1986 Sodium transport by rat cortical collecting tubule. Effects of vasopressin and desoxycorticosterone. J. Clin. Investig.; 77:1291-1298. 2420830 10.1172/JCI112433 PMC424479

[b52] Roger F. Martin P. Y. Rousselot M. Favre H. Feraille E. 1999 Cell shrinkage triggers the activation of mitogen‐activated protein kinases by hypertonicity in the rat kidney medullary thick ascending limb of the Henle's loop. Requirement of p38 kinase for the regulatory volume increase response. J. Biol. Chem.; 274:34103-34110. 10567379 10.1074/jbc.274.48.34103

[b53] Ron D. Walter P. 2007 Signal integration in the endoplasmic reticulum unfolded protein response. Nat. Rev. Mol. Cell Biol.; 8:519-529. 17565364 10.1038/nrm2199

[b54] Roth I. Leroy V. Kwon H. M. Martin P. Y. Feraille E. Hasler U. 2010 Osmoprotective transcription factor NFAT5/TonEBP modulates nuclear factor‐kappaB activity. Mol. Biol. Cell; 21:3459-3474. 20685965 10.1091/mbc.E10-02-0133 PMC2947481

[b55] Rutkowski D. T. Kaufman R. J. 2004 A trip to the ER: coping with stress. Trends Cell Biol.; 14:20-28. 14729177 10.1016/j.tcb.2003.11.001

[b56] Sands J. M. Layton H. E. 2014 Advances in understanding the urine‐concentrating mechanism. Annu. Rev. Physiol.; 76:387-409. 24245944 10.1146/annurev-physiol-021113-170350

[b57] Staub O. Gautschi I. Ishikawa T. Breitschopf K. Ciechanover A. Schild L. 1997 Regulation of stability and function of the epithelial Na+ channel (ENaC) by ubiquitination. EMBO J.; 16:6325-6336. 9351815 10.1093/emboj/16.21.6325 PMC1170239

[b58] Tian W. Cohen D. M. 2002 Urea stress is more akin to EGF exposure than to hypertonic stress in renal medullary cells. Am. J. Physiol. Renal. Physiol.; 283:F388-F398. 12167588 10.1152/ajprenal.00031.2002

[b59] Tomita K. Pisano J. J. Knepper M. A. 1985 Control of sodium and potassium transport in the cortical collecting duct of the rat. Effects of bradykinin, vasopressin, and deoxycorticosterone. J. Clin. Investig.; 76:132-136. 4019771 10.1172/JCI111935 PMC423727

[b60] Vehaskari V. M. Hempe J. M. Manning J. Aviles D. H. Carmichael M. C. 1998 Developmental regulation of ENaC subunit mRNA levels in rat kidney. Am. J. Physiol.; 274:C1661-C1666. 9611132 10.1152/ajpcell.1998.274.6.C1661

[b61] Verrey F. 1994 Antidiuretic hormone action in A6 cells: effect on apical Cl and Na conductances and synergism with aldosterone for NaCl reabsorption. J. Membr. Biol.; 138:65-76. 8189433 10.1007/BF00211070

[b62] Zentner M. D. Lin H. H. Deng H. T. Kim K. J. Shih H. M. Ann D. K. 2001 Requirement for high mobility group protein HMGI‐C interaction with STAT3 inhibitor PIAS3 in repression of alpha‐subunit of epithelial Na+ channel (alpha‐ENaC) transcription by Ras activation in salivary epithelial cells. J. Biol. Chem.; 276:29805-29814. 11390395 10.1074/jbc.M103153200

[b63] Zhang Z. Yang X. Y. Cohen D. M. 1999 Urea‐associated oxidative stress and Gadd153/CHOP induction. Am. J. Physiol.; 276:F786-F793. 10330061 10.1152/ajprenal.1999.276.5.F786

[b64] Zhang W. Xia X. Jalal D. I. Kuncewicz T. Xu W. Lesage G. D. 2006a Aldosterone‐sensitive repression of ENaCalpha transcription by a histone H3 lysine‐79 methyltransferase. Am. J. Physiol. Cell Physiol.; 290:C936-C946. 16236820 10.1152/ajpcell.00431.2005 PMC3009459

[b65] Zhang W. Xia X. Reisenauer M. R. Hemenway C. S. Kone B. C. 2006b Dot1a‐AF9 complex mediates histone H3 Lys‐79 hypermethylation and repression of ENaCalpha in an aldosterone‐sensitive manner. J. Biol. Chem.; 281:18059-18068. 16636056 10.1074/jbc.M601903200 PMC3015183

[b66] Zhang W. Xia X. Reisenauer M. R. Rieg T. Lang F. Kuhl D. 2007 Aldosterone‐induced Sgk1 relieves Dot1a‐Af9‐mediated transcriptional repression of epithelial Na+ channel alpha. J. Clin. Investig.; 117:773-783. 17332896 10.1172/JCI29850 PMC1804379

[b67] Zhou H. X. Rivas G. Minton A. P. 2008 Macromolecular crowding and confinement: biochemical, biophysical, and potential physiological consequences. Annu. Rev. Biophys.; 37:375-397. 18573087 10.1146/annurev.biophys.37.032807.125817 PMC2826134

